# Brazilian Society of Surgical Oncology: Guidelines and Consensus Statement for Palliative Surgery in Oncology

**DOI:** 10.1002/jso.70189

**Published:** 2026-01-07

**Authors:** Audrey Cabral Ferreira de Oliveira, Jordana Henz Hammes, Isabela Maria Alves de Almeida Oliva, Lara Andrade Mendes Mangieri, Ronald Enrique Delgado Bocanegra, Marcos Gonçalves Adriano Junior, Ana Caroline Fonseca Alves, Eliel Oliveira de Araujo, Jairo Cerqueira de Almeida Teixeira, Patricia Isabel Bahia Mendes Freire, Larissa de Jesus Almeida, Raquel Lacerda Dantas de Farias, Heládio Feitosa e Castro Neto, Alexandre Ferreira Oliveira, Reitan Ribeiro, Rodrigo Nascimento Pinheiro

**Affiliations:** ^1^ Oncoclínicas Salvador Brazil; ^2^ Pontifical Catholic University of Rio Grande do Sul – Graduate Program in Pediatrics and Child Health Porto Alegre Brazil; ^3^ High‐Complexity Oncology Unit Irmã Dulce Social Works Salvador Brazil; ^4^ School of Medicine, Hospital das Clínicas University of São Paulo São Paulo Brazil; ^5^ Department of Oncologic Surgery Santa Casa de Misericórdia da Bahia Salvador Brazil; ^6^ Oncologic Surgery – MedSênior Bethesda Maryland USA; ^7^ Hospital São Domingos São Luís Maranhão Brazil; ^8^ Federal University of Juiz de Fora Juiz de Fora Minas Gerais Brazil; ^9^ Surgical Oncology Department Oncoclinicas São Paulo Brazil; ^10^ State University of Pará (UEPA); Oncologic Surgeon Hospital Ophir Loyola Belém Brazil; ^11^ Santa Izabel Hospital Salvador Brazil; ^12^ Integra Oncology Institute São Paulo Brazil; ^13^ Surgical Oncology Service Cancer Institute of Ceará Fortaleza Brazil; ^14^ Surgical Oncology Service Federal University of Juiz de Fora Juiz de Fora Brazil; ^15^ Surgical Oncology Service Erasto Gaertner Hospital Curitiba Brazil; ^16^ Surgical Oncology Department Hospital de Base of the District Federal Brasília Brazil

**Keywords:** palliative care, palliative surgery, quality of life, surgical oncology

## Abstract

**Background and Objective:**

Palliative surgery in oncology aims to relieve symptoms, improve quality of life, and respect patient autonomy in advanced cancer. This study aimed to develop evidence‐based recommendations for safely indicating and performing palliative surgeries in Brazil, considering clinical, ethical, and multidisciplinary aspects.

**Methods:**

A modified Delphi consensus was conducted with nine experts from the Brazilian Society of Surgical Oncology, including surgical and clinical oncologists, palliative care specialists, and a psychologist. Sixteen key recommendations were formulated based on literature review and a national survey identifying gaps in training, communication, and technical safety. Consensus was defined as ≥ 80% agreement, achieved in a single round.

**Results:**

Recommendations emphasize individualized patient selection based on functional status, frailty, prognosis, and symptom severity. Multidisciplinary evaluation, shared decision‐making, clear communication, and consideration of minimally invasive techniques were prioritized. Palliative procedures focus on symptom control rather than survival extension, with evidence supporting improved quality of life, reduced hospital admissions, and enhanced oral intake.

**Conclusions:**

Palliative surgery should be guided by strict clinical criteria, multidisciplinary planning, and patient‐centered communication. Active patient participation, ethical deliberation, and evidence‐based practices ensure safe, effective, and humanized care, avoiding futile or disproportionate interventions.

## 1 Introduction

Palliative care is an approach that improves quality of life for patients and families facing serious illnesses, starting at diagnosis and often combined with life‐prolonging therapies [1, 2]. Surgical procedures are part of this approach, helping in diagnostic clarification, symptom relief, functional rehabilitation, and management of complications such as obstructions, fistulas, and hemorrhages [3].

Indicating palliative surgery is complex and requires evaluating prognosis, functional status, patient expectations, and potential impact on quality of life [4]. Multidisciplinary planning involving surgery, anesthesia, intensive care, and palliative care improves outcomes and reduces postoperative mortality [5].

Among hospitalized patients, palliative surgery accounts for up to 40% of surgical consultations, with a median survival of 2.9 months [6]. These procedures are frequent near the end of life: approximately 20% of patients undergo surgery in their last year, yet only 4%–38% receive palliative care beforehand [7]. In large centers, up to 20% of oncological surgeries are non‐curative, especially in gastrointestinal and gynecological cancers. Despite the potential for symptom relief, heterogeneity in indications and limited evidence regarding quality of life and survival hinder standardization, underscoring the need for clear technical and ethical criteria aligned with patient goals [8, 9, 10].

In Brazil, data on surgical approaches in advanced cancer are scarce. A National Cancer Institute study (2008) reported 174 procedures in 136 patients, mostly for head and neck (61.8%), cervical (16.2%), esophageal (9%), and lung (7%) cancers. Average survival was 90.1 days, with better outcomes in patients with Karnofsky index above 50%. Another series in unresectable periampullary tumors with jaundice showed longer survival with surgical biliary‐digestive diversion compared to endoscopic stenting (586 vs. 56 days) and fewer readmissions.

Although research is expanding, the absence of standardized guidelines for integrating surgery and palliative care contributes to variability, uncertainty, and inconsistent outcomes. Developing national consensus protocols is therefore essential to guide indication, execution, and follow‐up of oncological surgeries in palliative settings [10, 11].

## 2 Definition of the Problem

A national survey conducted by the Palliative Care Committee of the Brazilian Society of Surgical Oncology (SBCO) and presented at ASCO 2025, with 184 oncology surgeons from all regions of Brazil, revealed major gaps in training and practice: 91.9% reported insufficient preparation in palliative care, 69.6% rarely participated in courses, and 61.4% were uncertain about the impact of surgery on patients' quality of life. Additional barriers included limited resources (37.5%), lack of formal training (27.7%), communication difficulties (21.7%), and ethical dilemmas (11.4%). Despite most surgeons (95.7%) recognizing the importance of early integration of palliative care, only 55% had regular access to specialized teams, reinforcing the need for national protocols to promote training, technical safety, and standardized decision‐making [12].

## 3 Materials and Methods

### 3.1 Study Design

**Figure 1:**
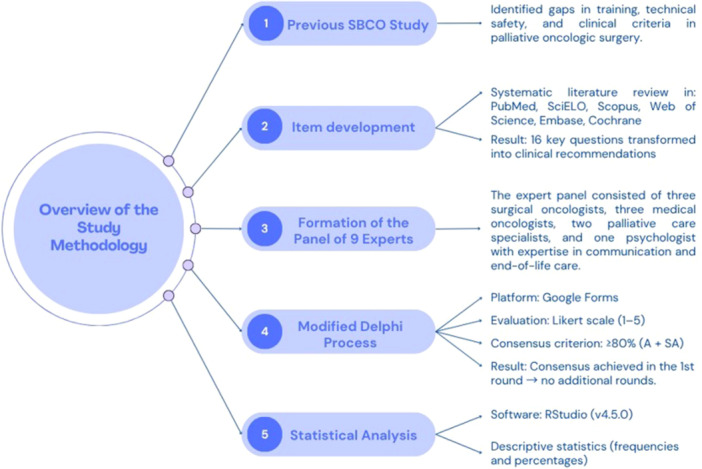
Overview of the study methodology used to develop the Brazilian Society of Surgical Oncology consensus on palliative surgery in oncology.

A consensus study using the modified Delphi method was conducted to develop practical recommendations in oncologic surgery within the context of palliative care in Brazil. An overview of the study methodology is presented in Figure 1.

The process was guided by findings from the study “Brazilian Society of Surgical Oncology: Realities and Challenges of Surgical Oncologists in the Practice of Palliative Care”, presented at ASCO 2025, which identified significant gaps in training, technical safety, and clinical practice criteria in this field [12].

### 3.2 Item Development

Between January and June 2025, a multidisciplinary committee of experts from the Brazilian Society of Surgical Oncology (SBCO) critically reviewed the results of the previous study as well as the relevant scientific literature, with searches conducted in PubMed (MEDLINE), SciELO, Scopus, Web of Science, Embase, and the Cochrane Library, prioritizing studies with moderate to high levels of evidence. From this analysis, 16 key questions were formulated and organized into clinical recommendations to be evaluated through consensus.

### 3.3 Expert Panel

The panel consisted of nine members of the SBCO Palliative Care Committee, including three surgical oncologists, three clinical oncologists (two with formal training in palliative care), two palliative care specialists, and one psychologist with expertise in clinical communication and end‐of‐life care. All participants formally accepted the invitation, declared no conflicts of interest, and authorized the anonymous use of their responses.

### 3.4 Delphi Procedure

A modified Delphi process was implemented using an online platform (Google Forms). Each recommendation was evaluated on a five‐point Likert scale: Strongly Disagree (SD), Disagree (D), Neither Agree nor Disagree (N), Agree (A), and Strongly Agree (SA).

The consensus criterion was predefined as ≥ 80% agreement, calculated as the sum of the “Agree” and “Strongly Agree” responses. Participants were also able to provide qualitative comments to justify their responses or suggest editorial adjustments.

The process was initially planned for up to three rounds. However, all items met the predefined consensus threshold during the first round. Therefore, no additional rounds were necessary. The qualitative feedback received was analyzed and used only for wording adjustments of the recommendations, without substantial changes to their content.

### 3.5 Evidence Grading

The recommendations were categorized according to the level of evidence and grade of recommendation following the Infectious Diseases Society of America (IDSA) system, adapted to the context of palliative oncologic surgery. The levels of evidence (Table 1) and grades of recommendation (Table 2) were used by the committee to classify each recommendation based on literature review and panel consensus.

**Table 1:** Levels of scientific evidence used for the development of the recommendations.

Level	Description
I	Evidence from at least one large randomized controlled trial (RCT) with low risk of bias or meta‐analyses of well‐conducted RCTs without heterogeneity
II	Small RCTs or large RCTs with potential bias, meta‐analyses of these trials, or RCTs with sample heterogeneity
III	Prospective cohort studies
IV	Retrospective cohort studies or case‐control studies
V	Studies without control groups, case reports, or expert opinion

*Note:* Classification of scientific evidence from strongest (I) to weakest (V).

**Table 2:** Grades of recommendation according to strength of evidence, clinical benefit, and risk.

Grade	Description
A	Strong evidence of efficacy with significant clinical benefit; strongly recommended
B	Strong or moderate evidence of efficacy but limited clinical benefit; usually recommended
C	Insufficient evidence of efficacy or benefits do not clearly outweigh risks; recommended in some cases
D	Moderate evidence of ineffectiveness or potential harms; rarely recommended
E	Strong evidence of ineffectiveness or high risk of harm; not recommended

*Note:* Strength of recommendation based on evidence, clinical benefit, and risk.

### 3.6 Statistical Analysis

The degree of recommendation was calculated using RStudio (version 4.5.0). Descriptive statistics were applied to summarize the strength of recommendations, and results were presented as frequencies and percentages.

## 4 Concept and Objectives

### 4.1 What Characterizes a Palliative Surgery in the Oncologic Context?

In oncology, palliative surgery focuses on relieving or preventing symptoms from tumor progression, without expectation of altering disease course or survival [13]. It must respect patient dignity and autonomy, ensuring the right to accept or refuse interventions, and relies on empathetic communication to define goals of care and avoid disproportionate treatments [14]. Beyond the surgical act, it includes psychological, social, and spiritual support, reinforcing the importance of discouraging procedures unlikely to meet patient goals and maintaining continuity of care when curative options are no longer feasible.

The concept of “Surgical Palliative Care” better reflects current practice, as it encompasses comprehensive management: rigorous control of physical symptoms (such as pain, nausea, or dyspnea), effective dialogue between professionals, patients, and families, and integration of psychosocial and spiritual support. This broader perspective positions surgery as one therapeutic tool within a multidisciplinary strategy aimed at alleviating suffering, preserving dignity, and promoting more humanized care [15].

**Recommendation 4.1**. Surgical palliative care should be implemented as a comprehensive, patient‐centered approach that encompasses effective communication with patients and families, multidisciplinary support (psychological, social, and spiritual), rigorous symptom control, and collaborative decision‐making that respects patient dignity, autonomy, and treatment goals rather than focusing solely on surgical intervention.

**Level of evidence:** V

**Grade of recommendation:** A

**Consensus level:** agreement — 100%; disagreement — 0%; voting abstention — 0%.

### 4.2 What Are the Main Objectives of Palliative Surgery in Oncology (Symptom Control, Improvement of Quality of Life, Prevention of Complications)?

Palliative surgery in oncology is a care intervention centered on active patient and family participation, ensuring that values and preferences guide decisions for symptom control, quality of life improvement, or complication prevention [16, 17]. Its main goal is symptom relief, with survival extension as a secondary objective [18, 19]. When carefully indicated, and supported by effective communication, palliative surgery can provide significant benefits, with reported success rates of 80%–90% in improving quality of life [5, 16, 20].

Indications vary by tumor site: in the upper gastrointestinal tract, pain accounts for 10%, bleeding and jaundice 35%, gastric obstruction 40%, and dysphagia 60%. In the lower tract, bleeding, perforations, and fistulas comprise 5%–10%, while obstruction represents 80%. In gynecological cancers, bleeding is 40%, obstructions and rectovaginal fistulas about 30% each [21]. Ultimately, value is defined by balancing clinical gains with risks and costs, highlighting the need for individualized strategies [22, 23, 24].

**Recommendation 4.2:** Palliative surgery should be offered only after thorough multidisciplinary evaluation and shared decision‐making that ensures patients and families understand the goals of symptom relief, potential risks and benefits, and alternatives, with priority given to those likely to achieve meaningful quality‐of‐life improvements rather than survival extension.

**Level of evidence:** III

**Grade of recommendation:** A

**Consensus level:** agreement — 99%; disagreement — 0%; voting abstention — 1%.

## 5 Criteria and Indications

### 5.1 Which Clinical and Prognostic Criteria Should be Considered Before Indicating a Palliative Surgery?

Before recommending palliative surgery in oncology, it is essential to carefully assess the patient's clinical status and prognosis using scores divided into three main groups. The first group evaluates functional status: the Karnofsky Performance Status (KPS) suggests that patients with ≥ 50% can tolerate palliative surgery, while scores < 40% indicate high risk [25, 26]. The Palliative Performance Scale (PPS), similar to KPS, shows limited surgical benefit when ≤ 30%–40% [27]. The ECOG scale, ranging from 0 to 5, considers surgery more feasible in patients with ECOG ≤ 2 [28].

The second group assesses frailty and prognosis. The Clinical Frailty Scale (CFS) and Fried Frailty Index identify patients at higher risk of complications when indicating moderate or advanced frailty [26, 28]. The Prognostic Nutritional Index (PNI) < 40 indicates severe malnutrition and poorer surgical outcomes [29].

Finally, the third group uses palliative prognostic scores, such as the PaP Score and PPI, which indicate short survival (< 30 days and < 3 weeks, respectively), discouraging surgery [27, 30]. The Glasgow Prognostic Score (GPS) also helps estimate survival in advanced cancer [29]. Surgical decisions should always involve multidisciplinary and family discussions to align expectations and avoid futile interventions [30].

**Recommendation 5.1:** Before recommending palliative surgery, systematically assess the patient's functional status, frailty, and prognosis using validated scoring systems. Procedures should be withheld in cases of very poor prognosis or high risk of complications, and decisions should be made through multidisciplinary discussion with family involvement.

**Level of evidence:** IV

**Grade of recommendation:** C

**Consensus level:** agreement — 92.16%; disagreement — 1.96%; voting abstention — 5.88%.

### 5.2 How to Differentiate a Palliative Surgery From a Cytoreductive or Curative Surgery?

We define palliative surgery in oncology as any surgical procedure whose primary goal is to alleviate or control a pathology or complication arising from an active oncological disease or its treatment, whether surgical or otherwise [31]. It differs from cytoreductive or curative surgeries in that its focus is on resolving a specific problem that causes symptoms, without necessarily addressing the tumor itself or the organ affected by cancer [32]. Palliative procedures are typically shorter, less invasive, and involve faster recovery, often favoring technically simpler and less complex surgeries [33].

In contrast, surgeries with curative intent involve more prolonged, complex, and sometimes higher‐risk procedures, as their primary objective is the complete removal of all tumor foci and cancer‐affected areas [34].

**Recommendation 5.2:** Palliative surgery should be defined as any procedure whose primary goal is symptom relief or control of complications in oncology patients, regardless of tumor eradication. This should be distinguished from curative or cytoreductive surgeries, which aim to completely remove tumor tissue and typically involve greater complexity and risk.

**Level of evidence:** I

**Grade of recommendation:** A

**Consensus level:** agreement — 97.06%; disagreement — 0.98%; voting abstention — 1.96%.

### 5.3 When Should Palliative Surgery Not be Indicated?

Main reasons to avoid palliative surgery include low symptom severity adequately managed clinically, patient preference, and high risk of complications [35]. Factors predicting shorter overall survival—such as poor performance status (ECOG > 2), fatigue, prior radiation, diffuse carcinomatosis, small bowel obstruction, anemia (Hb < 10.5), hypoalbuminemia (< 3.5), elevated CRP or creatinine, leukocytosis with neutrophilia, recent weight loss > 5 kg, and age > 65—also argue against surgery [36, 37, 38]. Extensive disease, including carcinomatosis, sarcomatosis, or dissemination at more than two sites, as well as ascites, hematologic malignancies, and neutropenia, further inform this decision [35, 36, 37, 38, 39].

Surgical indication should consider outcomes beyond overall survival, prioritizing symptom relief duration and quality of life improvement. Morbidity and mortality associated with the procedure, including operative time, length of hospital stay, need for reintervention, complication risk, and cost, must also be carefully evaluated to ensure that the intervention is both safe and beneficial for the patient [18, 40].

**Recommendation 5.3:** Avoid recommending palliative surgery for patients whose symptoms are manageable clinically, who prefer conservative treatment, or who have a high risk of complications. Poor functional status, abnormal laboratory markers, and advanced disease progression are factors associated with reduced survival and may contraindicate the procedure. Decisions should consider not only survival but also quality of life, surgical risks, and cost.

**Level of evidence:** III

**Grade of recommendation:** B

**Consensus level:** agreement — 78.44%; disagreement — 8.82%; voting abstention — 12.74%.

### 5.4 What Are the Indications and Benefits of Palliative Surgery for Intestinal Obstruction in Gastrointestinal Cancer?

The management of malignant bowel obstruction in gastrointestinal cancer prioritizes symptom relief, targeting nausea, vomiting, pain, and inability to eat, while considering serious complications like perforation or sepsis [41, 42]. Palliative surgery is indicated for patients with advanced disease who have refractory symptoms, failure of clinical management, or ineffectiveness of alternatives such as endoscopic therapies, which may reduce morbidity by up to 12% and should be considered first‐line [41, 43].

Palliative surgery can improve symptom control, restore oral intake, enhance nutritional status, reduce hospital admissions, and improve quality of life for patients and caregivers [44]. Careful patient selection, considering functional status, nutritional adequacy, and life expectancy, is essential to maximize benefits and minimize risks, including sepsis, fistulas, thromboembolism, and high readmission rates (up to 46%) [45, 46]. Minimally invasive approaches, such as laparoscopic or robotic surgery, offer reduced surgical trauma, faster recovery, and better pain control, making them especially valuable for patients with limited functional reserve [47].

**Recommendation 5.4:** Palliative surgery for malignant bowel obstruction should be prioritized only in cases of refractory symptoms and failure of clinical or endoscopic treatment, and after a favorable multidisciplinary evaluation. Less invasive methods should be preferred whenever available.

**Level of evidence:** I

**Grade of recommendation:** A

**Consensus level:** agreement — 92.16%; disagreement — 2.94%; voting abstention — 4.90%.

### 5.5 When to Indicate Palliative Urinary Diversion in Urological Tumors?

There is evidence that urinary diversion prevents deterioration of renal function and may improve survival in oncology patients [48, 49]. Urinary obstruction should be treated in cases of infection with fever, regardless of disease stage or expected survival [50]. Patients receiving palliative treatment may benefit from a reduction in creatinine after diversion, allowing continuation of medical therapy [51, 52, 53].

In obstructions causing potentially fatal renal failure, diversion should be individualized, preferably in a multidisciplinary context, taking into account prognosis, quality of life, and patient preference [50]. In terminal cancer, diversion can prolong survival by weeks or months, but may reduce quality of life due to pain, fatigue, and sequelae of advanced cancer [54].

There are several options for urinary diversion, including retrograde diversion, vesical diversion, ureterostomies, nephrostomies, and ileal conduits, with no consensus on the ideal method [55]. Retrograde diversion with a double‐J stent is frequently the first‐line approach, as it is less invasive, well tolerated, and has a high success rate, requiring periodic stent changes every 3 to 12 months. Tumor infiltration may necessitate more frequent stent changes or nephrostomy [56, 57, 58, 59, 60, 61].

Percutaneous nephrostomy is indicated for ureteral obstructions associated with cervical, prostate, or rectal cancer, bladder invasion from advanced prostate or bladder cancer, and in peritoneal carcinomatosis where pelvic mobilization is limited [62, 63, 64]. However, it significantly impacts quality of life, often requiring prolonged hospitalization, and in some cases, patients may never be discharged [65].

**Recommendation 5.5:** Palliative urinary diversion should be recommended only when there is clear potential clinical benefit, such as prevention of severe infection, preservation of renal function, or continuation of oncologic treatment, with priority given to less invasive methods whenever feasible.

**Level of evidence:** III

**Grade of recommendation:** A

**Consensus level:** agreement — 96.08%; disagreement — 1.96%; voting abstention — 1.96%.

### 5.6 What Is the Role of Palliative Surgery in Controlling Hemorrhage in Advanced Tumors?

Palliative surgery plays a critical role in managing hemorrhage in advanced tumors, which can severely compromise quality of life and, in some cases, be fatal. While clinical measures, systemic oncologic therapies, and minimally invasive interventions like radiotherapy, ablation, and embolization are often used, they may not always control bleeding [66]. Surgical intervention aims to stop blood loss, provide symptomatic relief, and prevent complications, directly improving patient functionality and quality of life [67].

Patient selection is challenging due to fragility, malnutrition, poor performance status, high postoperative morbidity and mortality, symptom recurrence, and limited life expectancy [68]. Decisions should involve multidisciplinary discussion, considering the patient's values, goals, and symptom management [68, 69]. Surgical techniques depend on tumor location, disease extent, clinical condition, and treatment objectives, ranging from partial or total tumor resection, bypasses, vascular ligation, to procedures like colostomy, ileostomy, gastrectomy, or palliative nephrectomy [70]. When conservative approaches fail, palliative surgery is essential for symptom control and clinical stabilization.

**Recommendation 5.6:** Palliative surgery for hemorrhage control in advanced tumors should be considered only when clinical and minimally invasive measures fail, with priority given to multidisciplinary evaluation and careful patient selection.

**Level of evidence:** III

**Grade of recommendation:** A

**Consensus level:** agreement — 92.16%; disagreement — 3.92%; voting abstention — 3.92%.

### 5.7 In Which Cases Is Gastrostomy or Jejunostomy Indicated for Palliative Feeding?

Palliative gastrostomy or jejunostomy is indicated when oral intake is impossible or unsafe, providing an alternative nutritional route to improve patient quality of life and support specific therapies. Gastrostomy is commonly used in patients with oropharyngeal tumors that prevent oral feeding or esophageal cancer obstructing gastrointestinal passage, while jejunostomy is preferred when gastric feeding is not feasible due to obstruction location [71]. Decisions should consider risks, benefits, and patient and family expectations, ideally involving a palliative care or clinical ethics team [72, 73]. Nutritional therapy plays a secondary role in end‐of‐life care, and alternative approaches, such as subcutaneous hydration, should be offered. Family support and education are essential, particularly regarding physiological changes such as anorexia‐cachexia and reduced gastrointestinal absorption [72].

Absolute contraindications include active peritonitis, uncorrectable coagulopathy, and ongoing intestinal ischemia, while relative contraindications include hemodynamic instability, respiratory compromise, ascites, anatomical alterations, and morbid obesity [74, 75, 76, 77, 78]. These factors must guide careful patient selection to maximize benefit and minimize procedural complications.

**Recommendation 5.7:** Palliative gastrostomy or jejunostomy should be recommended only for patients without absolute contraindications, when oral intake is not possible or safe, and when there is a realistic expectation of improved quality of life, following thorough discussion of risks and benefits with the patient and/or family.

**Level of evidence:** II

**Grade of recommendation:** A

**Consensus level:** agreement — 94.12%; disagreement — 0.98%; voting abstention — 4.90%.

### 5.8 When Are Thoracentesis and Surgical Pleurodesis Indicated for Malignant Pleural Effusions?

Malignant pleural effusions (MPEs) affect about 15% of cancer patients, most commonly those with advanced lung or breast tumors, causing dyspnea, chest pain, and cough, with significantly impaired quality of life and limited survival (3–12 months) [79, 80, 81]. When MPEs are refractory to systemic or symptomatic treatment, invasive interventions are considered, guided by symptom severity, patient condition, life expectancy, underlying disease, and effusion volume, aiming for durable symptom relief and lung reexpansion (5).

Thoracentesis is initially indicated to confirm malignancy, provide immediate symptomatic relief, and evaluate response to fluid drainage (1, 2). Surgical pleurodesis, inducing adhesion between visceral and parietal pleura, prevents recurrence and is recommended for patients with life expectancy over 1 month, performance status ≤ 3, lung reexpansion after initial drainage, and no bronchial obstruction or lung entrapment (1, 2, 6, 7). If reexpansion fails, alternatives include indwelling pleural catheters, repeated thoracenteses, or pleuroperitoneal shunts (1, 8).

**Recommendation 5.8:** Invasive intervention for symptomatic malignant pleural effusion should be considered only when clinical treatment is refractory, prioritizing less invasive methods that provide effective symptom control, such as thoracentesis, pleurodesis, or indwelling pleural catheter placement, based on the patient's clinical condition and life expectancy.

**Level of evidence:** II

**Grade of recommendation:** B

**Consensus level:** agreement — 94.12%; disagreement — 0.98%; voting abstention — 4.90%.

### 5.9 How Can Palliative Biliary Diversion, Surgical, Percutaneous, or Endoscopic, Benefit Patients With Obstructive Cholestasis Due to Pancreatic or Biliary Cancer?

Obstructive cholestasis is common in patients with advanced pancreatic or biliary cancer, causing jaundice, pruritus, cholangitis, and liver dysfunction, which negatively affect quality of life and eligibility for systemic therapies [82, 83, 84]. Palliative biliary diversion aims to restore bile flow and relieve symptoms, with the choice of procedure guided by life expectancy, tumor anatomy, and patient condition.

Endoscopic biliary drainage via endoscopic retrograde cholangiopancreatography (ERCP) is the preferred approach, with self‐expanding metal stents (SEMS) recommended for longer patency [85, 86]. Percutaneous biliary drainage (PBD) serves as an alternative in complex cases, such as hilar tumors, and advanced techniques like endoscopic ultrasound‐guided biliary drainage (EUS‐BD) are promising when ERCP fails [87, 88].

Surgical biliary bypass, including choledochojejunostomy, is indicated for patients with longer life expectancy and duodenal obstruction, offering durable drainage but with higher morbidity compared to ERCP [89, 90, 91, 92]. Overall, the choice between endoscopic, percutaneous, or surgical approaches should be individualized, prioritizing symptom relief and quality of life rather than survival [93, 94].

**Recommendation 5.9:** Palliative biliary drainage via the endoscopic route should be recommended as the first‐line approach, using self‐expanding metal stents whenever feasible. Percutaneous or surgical drainage should be considered only in selected cases, always prioritizing symptom relief and the patient's quality of life.

**Level of evidence:** II

**Grade of recommendation:** B

**Consensus level:** agreement — 94.12%; disagreement — 0.98%; voting abstention — 4.90%.

## 6 Multidisciplinary Approach

### 6.1 How Can the Multidisciplinary Team Assist in the Decision‐Making Process Regarding Palliative Surgery?

The involvement of a multidisciplinary team is essential in deciding whether to perform palliative surgery, as it ensures a comprehensive evaluation of the patient's physical, psychological, and social conditions. Beyond clinical aspects such as pain and symptom management, it is crucial to respect the patient's perception of illness, personal values, family and social relationships, and overall quality of life [67, 95, 96].

The team may include physicians, nurses, psychologists, social workers, physiotherapists, nutritionists, and occupational therapists, each contributing specific expertise. Physicians assess clinical status, nurses address care needs, psychologists provide emotional support, and social workers evaluate family and financial conditions. Other professionals are involved as necessary [97]. Decisions are typically reached in joint meetings, where benefits and risks are weighed collectively. The outcome is then shared with the patient and family, prioritizing well‐being, quality of life, and, most importantly, the patient's wishes [98, 99].

**Recommendation 6.1:** Before recommending palliative surgery, ensure a multidisciplinary evaluation that includes healthcare professionals, the patient, and the family, with priority given to quality of life, symptom relief, and respect for the patient's wishes.

**Level of evidence:** III

**Grade of recommendation:** A

**Consensus level:** agreement — 94.12%; disagreement — 0%; voting abstention — 5.88%.

### 6.2 What Strategies Can be Adopted to Ensure That the Patient and Their Family Understand the Benefits and Limitations of the Surgery?

Clear, empathetic communication is essential to ensure patients and their families understand the benefits, risks, and limitations of palliative surgery. This process allows them to make informed decisions that align with their values and expectations [100, 101].

Strategies include assessing the patient's and family's level of understanding about the disease and prognosis, avoiding excessive technical language, and explaining in an objective way the prognosis, possible complications, and realistic surgical outcomes. It is also important to provide detailed information about the nature of the surgery, potential adverse effects, and the likelihood of recurrence or disease progression, even in cases of poor prognosis [100, 101].

**Recommendation 6.2:** Ensure clear, objective, and empathetic communication with the patient and family before recommending palliative surgery, clarifying benefits, risks, limitations, and alternatives, and promoting shared decision‐making consistent with the patient's values and expectations.

**Level of evidence:** IV

**Grade of recommendation:** A

**Consensus level:** agreement — 97.06%; disagreement — 0.98%; voting abstention — 1.96%.

## 7 Postoperative Pain Control

### 7.1 What Are the Best Strategies for Pain Control in the Postoperative Period of Palliative Surgeries?

The approach should be integrated into a comprehensive perioperative care plan, including adequate anesthesia, multidisciplinary interventions, and physical therapy, which encompasses the concept of “total pain,” with ongoing postoperative care [102]. Adaptation to the type of surgery performed is important. Specific guidelines are available after abdominal, breast, and prostate cancer surgery [103, 104, 105].

The pharmacological approach should be multimodal, safe, effective, and continuously reassessed [106, 107]. Analgesic doses vary considerably between patients. These include simple analgesics and selective or nonselective NSAIDs administered preoperatively or intraoperatively and continued postoperatively [105]. Tricyclic antidepressants, SNRIs, such as duloxetine, and anticonvulsants, such as pregabalin, are used as adjuvants in neuropathic pain. Regional nerve blocks are used in situations with specific physical areas of pain, such as epidural catheters in those with fractures. Corticosteroids, such as dexamethasone, are used in metastatic bone disease, epidural spinal cord compression, tumor infiltration of a nerve, and visceral pain with bowel obstruction or bladder spasm in combination with anticholinergics, such as scopolamine [108, 109].

Strong opioids, such as morphine, oxycodone, methadone, and fentanyl, should be used, unless contraindicated, in those with moderate to severe pain at the lowest dose possible to achieve analgesia according to the patient's goals, in addition to postoperative rescue. In those with substance use disorder (SUD), a PC, pain, and/or SUD specialist should be consulted to determine the optimal approach [110, 111]. Side effects, including constipation, hypotension, sedation, respiratory depression, delirium, and other alterations in consciousness, should be monitored, and prevention and treatment strategies provided [111, 112].

The benefits of integrative medicine are highlighted by techniques such as acupuncture and music therapy. However, the overall quality of the evidence is low [113, 114].

**Recommendation 7.1:** Pain management in palliative oncologic surgery should be multimodal, incorporating opioids, NSAIDs, adjuvants, and regional blocks, with systematic monitoring of side effects. Complementary therapies may be considered, although current evidence of benefit is limited.

**Level of evidence:** I

**Grade of recommendation:** A

**Consensus level:** agreement — 99,02%; disagreement — 0%; voting abstention — 1%.

## 8 Effectiveness

### 8.1 How Can the Effectiveness of Palliative Surgery in Patients' Quality of Life be Assessed?

Palliative surgery primarily aims to improve quality of life, with symptom relief, especially pain control, being the main indicator for intervention [115]. Traditional outcomes such as morbidity, mortality, and disease recurrence are often insufficient to capture its true benefit. Studies suggest that about 40% of oncologic surgical procedures in large tertiary centers are performed with palliative intent, emphasizing the need for appropriate evaluation metrics [116]. Literature shows conflicting results regarding effectiveness due to the lack of standardized measures and variability across clinical contexts. For example, prospective studies report no quality‐of‐life improvement after surgery in gastric cancer patients with peritoneal carcinomatosis, despite better oral intake [117].

Conversely, other trials indicate enhanced quality of life in patients with gastric or gastroesophageal junction cancer, along with higher discharge rates and fewer readmissions [118]. Emerging evidence suggests that open‐ended questionnaires may better capture complex symptoms than structured surveys such as FACT‐G, offering a feasible strategy to align expectations and assess perioperative outcomes more accurately [116]. These approaches support individualized decision‐making and a more reliable evaluation of the effectiveness of palliative surgery.

**Recommendation 8.1:** The effectiveness of palliative surgery should be assessed primarily through symptom relief and improvement in quality of life, employing validated assessment tools and individualized evaluation approaches.

**Level of evidence:** II

**Grade of recommendation:** A

**Consensus level:** agreement — 91,18%; disagreement — 1.96%; voting abstention — 6.86%.

## 9 Decision‐Making and Ethical Challenges

### 9.1 What Are the Ethical Challenges Involved in the Decision‐Making Process Regarding the Performance of Palliative Surgeries?

The deliberation process for palliative surgery begins with a technical assessment of eligible patients, considering potential benefits such as symptom control, management of oncological complications, quality of life, prognosis, alternative clinical strategies, procedure complexity, and morbidity and mortality. A clear distinction between futile and technically inappropriate interventions must be made objectively, aligning with the central goal of care and balancing the principles of beneficence and autonomy [119, 120].

Shared decision‐making should be multidisciplinary, involving surgeons, oncologists, palliative care specialists, and the patient‐family unit, with assertive and empathetic communication strategies to create an individualized care plan. Understanding patient values, the meaning of suffering, and perception of disease and prognosis is essential to preserve autonomy and align expectations regarding outcomes and technical feasibility. Honest communication and respect for patient wishes are major ethical challenges, alongside managing unrealistic expectations, limited healthcare resources, and moral distress among healthcare professionals, a phenomenon sometimes referred to as “mistanásia” in Brazil [119, 120].

**Recommendation 9.1:** Ensure the patient's autonomy in jointly determining the goals of care through assertive communication about which interventions would be futile or technically inappropriate. Facilitate discussion of possible scenarios, with or without surgical interventions, while avoiding shifting responsibility or adopting a paternalistic approach in the decision‐making process.

**Level of evidence:** V

**Grade of recommendation:** A

**Consensus level:** agreement — 98,04%; disagreement — 1,96%; voting abstention — 0%.

## 10 Conclusion

Palliative surgery in oncology is a fundamental strategy for symptom relief, preserving quality of life, and respecting patient autonomy in advanced cancers. Indications should be based on strict clinical criteria, multidisciplinary evaluation, and clear, empathetic communication with patients and families, prioritizing individualized and proportional interventions. Decisions should be guided by realistic expectations regarding symptomatic benefit, risks, and functional impact, avoiding futile or technically inappropriate procedures. Active patient participation, objective selection criteria, attention to pain control and functionality, and adherence to the best available evidence ensure that palliative surgical care is safe, ethical, and humanized.

## Synopsis

This study presents evidence‐based recommendations for palliative surgery in oncology in Brazil, focusing on symptom relief, quality of life, and patient autonomy. Using a modified Delphi consensus, experts emphasized individualized patient selection, multidisciplinary evaluation, and shared decision‐making. The guidelines aim to ensure safe, effective, and ethical care while avoiding futile or disproportionate interventions.

## Data Availability

The data that support the findings of this study are available from the corresponding author upon reasonable request.
